# Peripheral ossifying fibroma: A 20-year retrospective study with focus on clinical and morphological features

**DOI:** 10.4317/medoral.25454

**Published:** 2022-06-19

**Authors:** Israel Leal Cavalcante, Caio César da Silva Barros, Vitória Maria Sousa Cruz, John Lennon Silva Cunha, Lara Cunha Carneiro Leão, Renata Roque Ribeiro, Eveline Turatti, Bruno Augusto Benevenuto de Andrade, Roberta Barroso Cavalcante

**Affiliations:** 1 Department of Dentistry, University of Fortaleza (UNIFOR), Fortaleza, CE, Brazil; 2 Department of Oral Diagnosis and Pathology, Postgraduate Program in Dentistry, School of Dentistry, Federal University of Rio de Janeiro (UFRJ), Rio de Janeiro, RJ, Brazil; 3 Department of Dentistry, Postgraduate Program in Dental Sciences, Federal University of Rio Grande do Norte (UFRN), Natal, RN, Brazil; 4 Department of Oral Diagnosis, Piracicaba Dental School, University of Campinas (UNICAMP), Piracicaba, SP, Brazil

## Abstract

**Background:**

Peripheral Ossifying Fibroma (POF) is a reactive hyperplastic lesion that exclusively occurs in the gingiva and is characterized by the deposition of dystrophic calcification, cementum-like tissue, and immature and mature bone within the connective tissue. The objective of the present study was to perform a retrospective analysis of clinicopathologic features of POF.

**Material and Methods:**

Clinical and histopathological data were obtained from biopsy records and histopathological reports from a Brazilian reference service in Oral Pathology (1999 - 2020). Morphological analysis was performed to evaluate features related to the mesenchymal component, inflammatory infiltrate, ulceration, and mineralized tissue.

**Results:**

A total of 270 POFs were diagnosed during the study period. A higher frequency was observed in females (71.9%) between the third (22.9%) and fourth (23.3%) decades of life. The anterior upper gingiva (29.1%) was the most affected region. Mature (86.7%) and immature (52.6%) bone tissue were the most frequent. There was a significant association between immature bone deposition and lesions with size ≤ 1.7 cm (*p* = 0.041); immature bone and cement-like tissue deposition with an evolution time ≤ 16 months (*p* < 0.001); deposition of immature bone and mesenchymal hypercellularization (*p* < 0.001); deposition of dystrophic calcification and the presence of ulceration (*p* < 0.001).

**Conclusions:**

The clinical characteristics corroborate the findings in the literature. The heterogeneous distribution and quantity of mineralized tissues found in the analyzed cases support the theory that the different mineralized tissues constitute a spectrum of clinical maturation of POF.

** Key words:**Gingiva, gingival diseases, gingival hyperplasia, oral pathology, diagnosis.

## Introduction

Peripheral ossifying fibroma (POF) is a common inflammatory/reactive lesion exclusively observed in the periodontal tissues. POF mostly affects the anterior maxilla of young adults and females, and local irritant factors such as trauma, dental biofilm, calculus, and irregular restorations have been associated with this lesion (1,2). Although POF pathogenesis remains unclear, it is believed that it may be originated from the gingival soft tissues or the periosteum as well as from the superficial periodontal ligament (3,4).

Clinically, POF arises as a gingival mass with a slow and progressive growing potential, generally without radiographic alterations, but radiopaque areas may be identified. The clinical appearance of POF resembles other gingival reactive lesions; thus, histopathological analysis is essential to the definitive diagnosis (1,2). POF is microscopically characterized by a highly cellular fibrous connective tissue composed of spindle-shaped and ovoid mesenchymal cells with vesicular nucleus and exhibit variable amounts of focal or extensive deposition of mature and/or immature bone, cementum-like tissue, and dystrophic calcification (3,4). Also, a variable amount of inflammatory infiltrate and presence of ulceration may be observed (1).

Due to the relatively high rate of recurrence (8% to 20%), this study may be of particular interest in periodontics since POFs exclusively affects the gingival tissues and may cause a significant post-surgical soft tissue cosmetic problem. Besides that, this study also provides a better understanding of this lesion and helps general dentists, periodontists, and oral pathologists in POF's diagnosis (4-6). In this context, the objective of this study was to analyze the clinicopathological features of POFs diagnosed in a referral oral and maxillofacial pathology diagnostic service of the Brazilian northeast, as well as evaluate the association between the clinical and histopathologic characteristics observed in this lesion.

## Material and Methods

- Study design

This retrospective cross-sectional clinicopathological study analyzed all cases of POF diagnosed in the Oral Pathology Service of UNIFOR (January 1999 to June 2020). Clinical data regarding sex, age, anatomic site, associated teeth, symptomatology, clinical aspect, insertion, lesion size, duration, radiographic characteristics, recurrence, and clinical diagnosis were collected from the clinical records and evaluated. On the other hand, cases without formalin-fixed paraffin-embedded sufficient material to perform the histopathological analysis were excluded.

- Morphological study

For morphological analysis, 5-µm sections were obtained from each case and stained with hematoxylin and eosin. POFs were evaluated under an optical microscope (Olympus CX31, Olympus Japan Co., Tokyo, Japan) by two previously calibrated examiners. Morphological features related to the mesenchymal cellularization, presence, type, and intensity of the inflammatory infiltrate, as well as the histopathological presence of ulceration, were evaluated.

The type and presence of mineralized tissue were evaluated semi-quantitatively adapting the methodology proposed by Lázare *et al*. (4). Thus, the mineralized tissue was classified as: mature bone when characterized as lamellar, organized, and stress-orientated bone; immature bone when exhibited woven osteoid type, random and non-stress-oriented; cementum-like tissue when basophilic acellular globules mineralized bodies was observed; and dystrophic calcifications when non-organized granular foci of calcifications were detected. Also, the presence of the mineralized tissue was graded as: - (absent), + (when it was present in < 10% of the lesion), ++ (10% to 50%), +++ (> 50%).

- Statistical analysis

Data were analyzed using the Statistical Package for the Social Sciences (SPSS 22.0; IBM Corp., Armonk, USA). Descriptive statistics was performed to the characterization of the sample. The weighted kappa coefficient was calculated to assess the interobserver morphological features agreement (≤ 0.20, slight agreement; 0.21 to 0.40, fair agreement; 0.41 to 0.60, moderate agreement; 0.61 to 0.80, good agreement; 0.81 to 1, excellent agreement). The non-parametric Mann-Whitney test with Bonferroni correction was performed verify the association between the types and presence of mineralized tissue (mature and immature bone, cementum-like tissue, and dystrophic calcification) and the clinicopathological features (anatomic site, size, duration, recurrence, mesenchymal cellularization, presence and intensity of inflammatory infiltrate, and histopathological presence of ulceration). Spearman’s correlation coefficient was performed to verify possible correlations among the different types of mineralized tissue (mature and immature bone, cementum-like tissue, and dystrophic calcification). For all tests, the *p* ≤ 0.05 was considered statistically significant.

## Results

- Clinical data

A total of 17,030 cases of oral and maxillofacial lesions were diagnosed in the analyzed period. Of these, 270 (1.5%) received the histopathological diagnosis of peripheral ossifying fibroma. As described in Table 1, a higher incidence of POFs was observed in female patients (n = 194; 71.9%; M:F = 1:2.5) in the third (20 - 29 years) (n = 58; 22.9%) and fourth (30 - 39 years) (n = 59; 23.3%) decades of life and was observed a mean age of 35.5 ± 15.9 years.

**Table 1:**
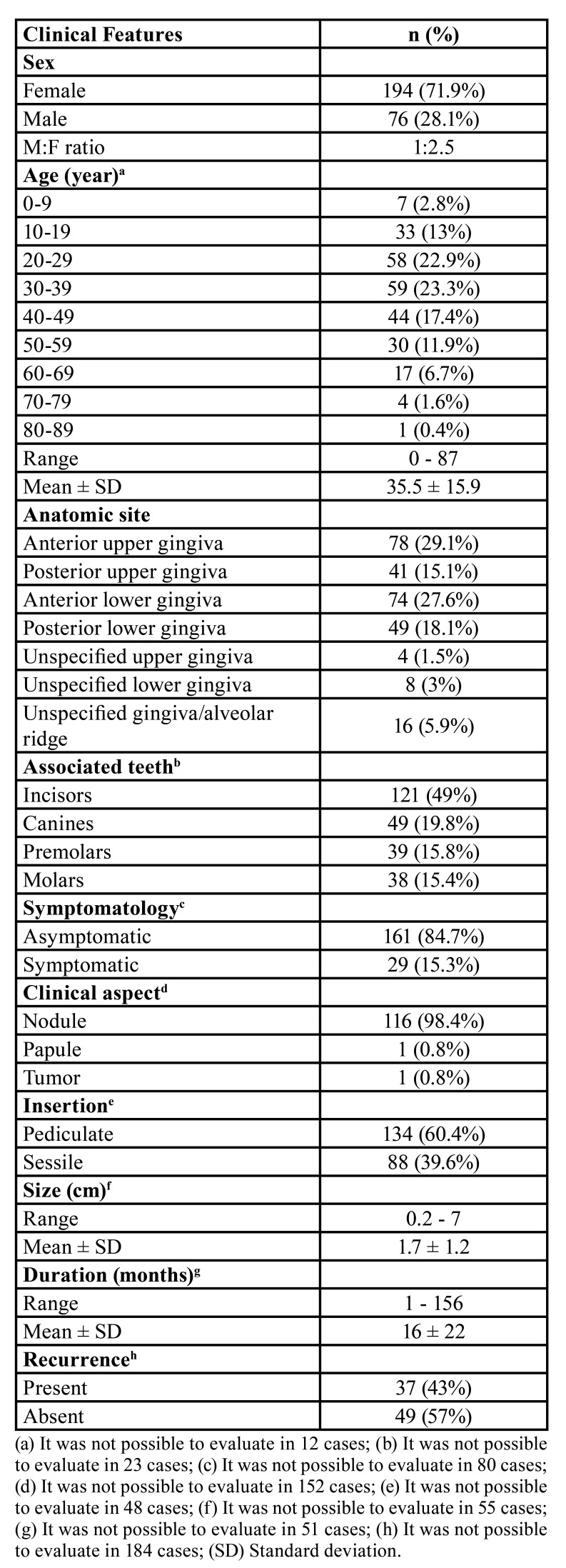
Relative and absolute frequency of the clinical features.

Regarding the anatomic site, the anterior upper gingiva was the most affected anatomical location (n = 78; 29.1%), followed by the anterior lower gingiva (n = 74; 27.6%) (Table 1). Also, it was observed that the incisors were the teeth mostly associated with POF (n = 121; 49%), followed by the canines (n = 49; 19.8%) (Table 1). Interestingly, it was observed two POF cases associated with neonatal incisors in a 4-months (tooth 61) and 5-months (tooth 71) old children.

**Figure 1:**
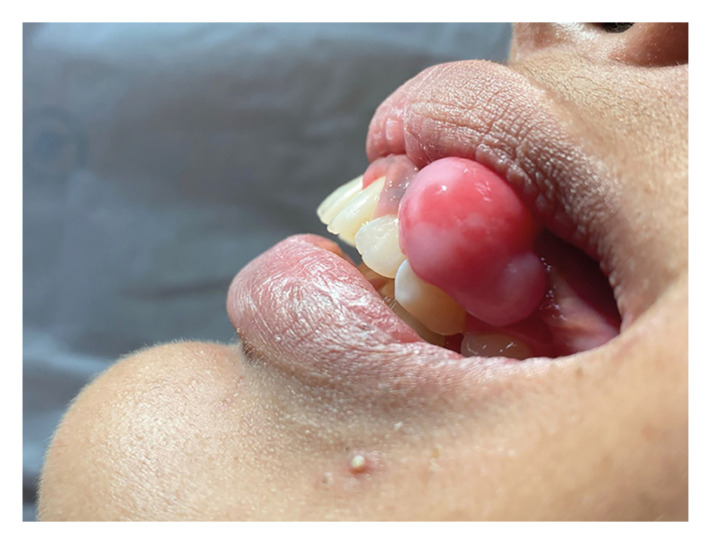
Clinical aspect of peripheral ossifying fibroma - Nodular and pedunculated lesion in anterior upper gingiva, exhibiting erythematous areas.

It was observed that 84.7% (n = 161) of the cases were asymptomatic. Also, 98.4% (n = 116) and 60.4% (n = 134) were characterized as a nodular and pedunculated lesion, respectively (Fig. 1). The lesions exhibited a mean size of 1.7 ± 1.2 cm (range: 0.2 - 7 cm) and a mean duration time of 16 ± 22 months (range: 1 - 156 months) (Table 1). Concerning the radiographic aspects, 26 (9.6%) cases showed radiographic findings. Of these, the presence of radiopaque foci and mild alveolar bone resorption was observed in 73.1% (n = 19) and 26.9% (n = 7) of cases, respectively.

Besides that, in 37 (43%) cases there was lesion recurrence (Table 1), of these there was one recurrence in 32 (86.5%) cases and three recurrences in 5 (13.5%) cases. Also, in only 20.4% (n = 55) of the cases analyzed the clinical hypothesis corresponded to the histopathological diagnosis. The following clinical hypotheses were cited: pyogenic granuloma (n = 123; 45.6%), fibroma (n = 27; 10.8%), peripheral giant cell granuloma (n = 20; 8%), fibrous hyperplasia (n = 10; 4%), gingival hyperplasia (n = 9; 3.6%) and congenital epulis (n = 1; 0.4%).

- Morphological data

**Figure 2:**
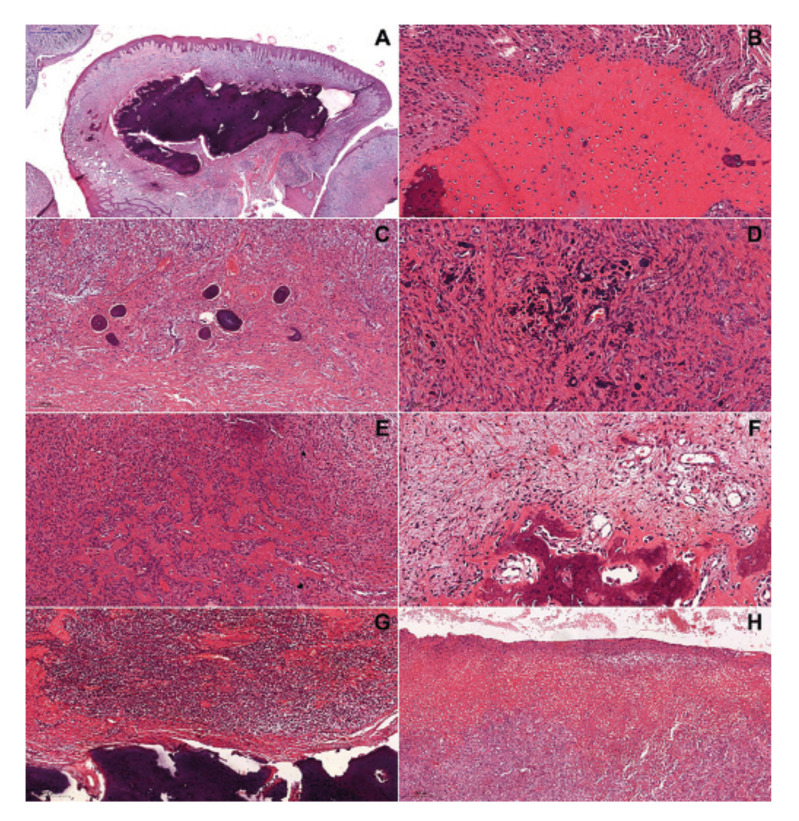
Histopathological features of the peripheral ossifying fibroma (hematoxylin and eosin) - Lesions showing deposition of (A - 1000 µm) mature bone, (B - 100 µm) immature bone, (C - 200 µm) cementum-like tissue, and (D - 50 µm) dystrophic calcification. Bone tissue deposition in (E - 100 µm) hypercellularized and (F - 50 µm) hypocellularized areas. Chronic inflammatory infiltrate close to mineralized tissue (G - 200 µm). Ulceration (H - 200 µm).

In the microscopic analysis, the agreement between the two examiners was excellent in the histopathological features analysis (weighted kappa coefficient 0.87). The presence of more than one type of mineralized tissue was observed in all analyzed cases (Fig. 2). Of these, mature and immature bone tissue were the most frequent (n = 234; 44.8%, and n = 142; 27.2%, respectively) (Table 2). Also, it was observed that the mesenchyme was hypercellularized in 161 (59.6%) cases (Table 3) (Fig. 2). Regarding the inflammatory infiltrate, it was present in 241 (89.3%) cases, of which 99.6% (n = 240) was of the chronic type and 54.8% (n = 54.8%) exhibited mild intensity (Table 3) (Fig. 2). In 94 (36.4%) cases was observed the histopathological presence of ulceration (Table 3) (Fig. 2).

Regarding the association between the mineralized tissue and the clinicopathological features, it was observed the lower gingiva significantly exhibits more mature bone tissue (*p* = 0.020) and lesions in the upper gingiva showed significantly more cementum-like tissue (*p* = 0.045). POFs with a size less than or equal to 1.7 cm exhibit a high significantly presence of immature bone tissue (*p* = 0.041), while lesions with a duration less than or equal to 16 months present a high significantly presence of immature bone tissue and cementum-like tissue (*p* < 0.001; *p* < 0.001, respectively). Also, POFs exhibiting a hypercellularized mesenchyme and presence of ulceration showed a significantly more presence of immature bone (*p* < 0.001) and dystrophic calcifications (*p* < 0.001), respectively.

**Table 2:**
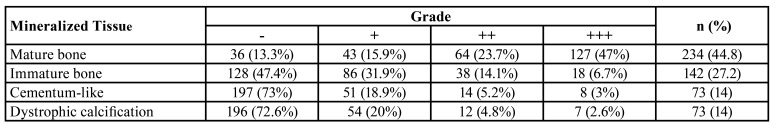
Relative and absolute frequency of the mineralized tissue.

**Table 3:**
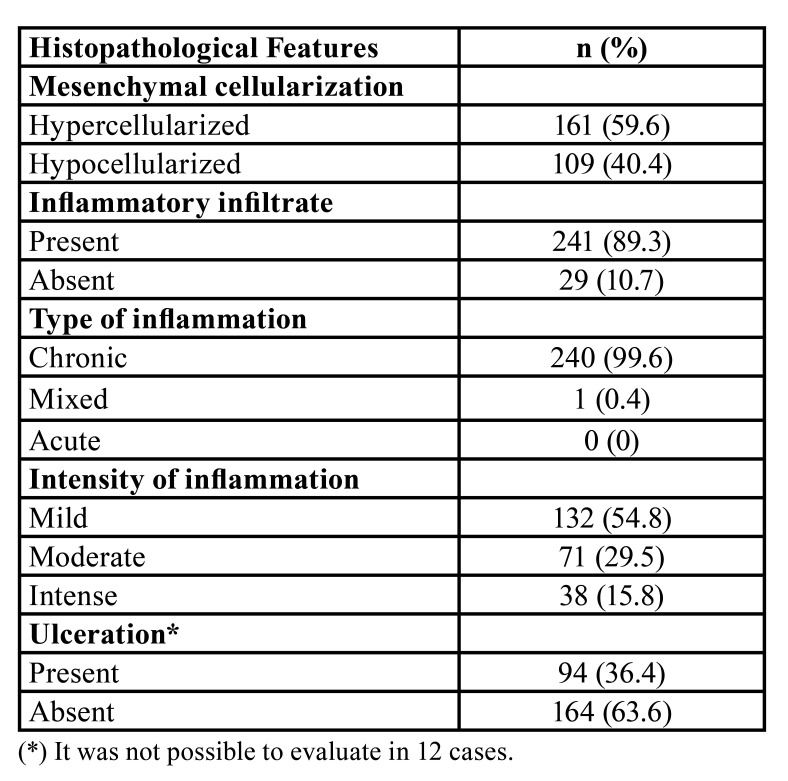
Relative and absolute frequency of the histopathological features.

- Correlation of the mineralized tissues

**Figure 3:**
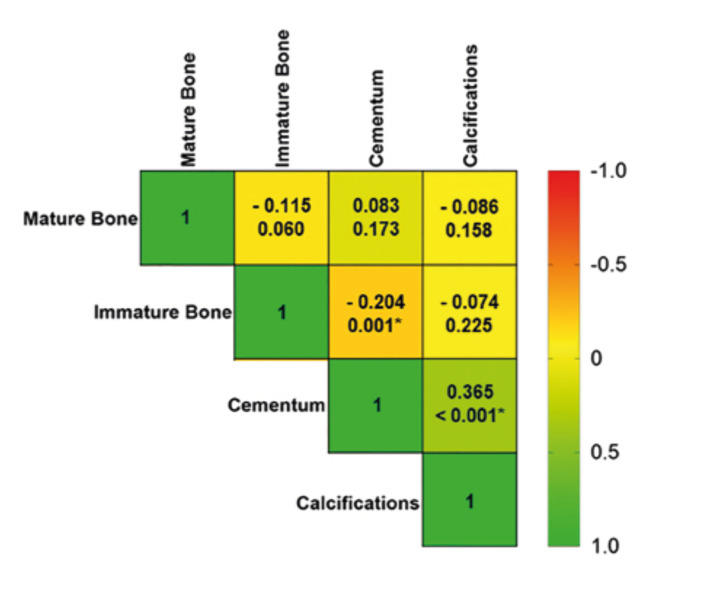
Spearman's rank correlation matrix among the type of mineralized tissues. Green represents a positive correlation and red is a negative correlation, as shown in the color key (* represents a statistically significant result).

The correlation results are presented in Fig. 3. A negative and statistically significant correlation between immature bone and cementum-like tissue (rho = - 0.204; *p* = 0.001) was observed. Also, there was positive and statistically significant correlation between cementum-like tissue and dystrophic calcification (rho = 0.365; *p* < 0.001).

## Discussion

Peripheral ossifying fibroma is an indolent lesion that exhibits clinical similarity to other gingival reactive lesions, making its clinical diagnosis a challenge. Also, its treatment can promote gingival aesthetic defects (2,7). This lesion was initially described by Shepherd *et al*. (8) as an alveolar exostosis, and later Eversol and Robin (9) proposed the term POF. However, it is also known as peripheral cemento-ossifying fibroma, ossifying fibro-epithelial polyp, peripheral fibroma with osteogenesis, peripheral fibroma with cementogenesis, peripheral fibroma with calcification, calcifying or ossifying fibroma epulis, and calcifying fibroblastic granuloma (6,8-10). To the best of our knowledge, this is the largest sample of POFs in clinicopathological research since the classic study carried out by Buchner and Hansen in 1987 (11). Our findings support the theory that the different mineralized tissues microscopically found in POF constitute a spectrum of maturation of this lesion.

It is known that POF exhibits a higher prevalence in the anterior upper gingiva, and it is mainly associated with the incisors and canines (12,13). Buchner *et al*. (14) performed one of the largest series of reactive hyperplastic lesions in the gingiva and found that the POF was more frequent in the anterior upper gingiva, corroborating the results of the present study, where 78 cases (28.9%) were in this anatomic site. Regarding the associated teeth, our results show that incisors were the teeth mostly associated with POF (n = 121; 49%), followed by the canines (n = 49; 19.8%), being this result similar to those presented in the literature (4).

From a clinical point of view, POF occurs more commonly in females (4,13,15). Similarly, the present study exhibited a higher frequency of this lesion in females, corresponding to a total of 71.9% of the analyzed cases. It is believed that the higher occurrence of POFs in this sex is associated with hormonal influences, especially estrogen and progesterone, once hormonal level variations can lead to changes in the production of gingival crevicular fluid (16,17). Besides that, it is important to note that the higher frequency of POF in females might be related to the first theory of the etiology of this lesion since hormonal changes are also a factor associated with the arise of pyogenic granuloma. In this context, this theory suggests that POF may initially appear as a pyogenic granuloma, which will later exhibit calcified tissue. However, the immunohistochemical analysis performed by Marcos *et al*. (18) does not demonstrate the expression of estrogen or progesterone receptors in the proliferating cells of POFs, which may indicate a low influence of these hormones in the arising of this lesion. These findings support the second theory of POF's etiology, which reports that this lesion arises as an inflammatory hyperplasia originating from the periodontal ligament cells. Also, the fact POF is a lesion that exhibits proximity to the periodontal ligament and occurs exclusively in gingival tissue makes this theory the most accepted nowadays (10,13,19).

In accordance with the literature (4,20), the present study showed a higher frequency of POFs in the third and fourth decades of life. However, it is important to highlight that this lesion occurs in a broad age range, including newborns, as shown in our results. Gingival lesions rarely occur in newborns, but when it is present, they often cause concern for parents, irritate babies, and impair feeding and breathing (21). The newborn lesions cases in this study occurred in the anterior maxilla, probably as a result of the presence of natal/neonatal teeth, which are more common in this region. As already mentioned, POF can arise from periodontal ligament cells, and the presence of these teeth can cause a low-grade local irritation and the development of this lesion (21,22).

Regarding the clinical features, POF usually presents as a slow-growing small nodular lesion, not exceeding 2 cm in diameter, asymptomatic, well-delimited, and with a pedunculated or sessile base (19,23,24). In line with the literary findings, we observed a nodular clinical presentation in 98.4% of the cases analyzed, as well as a higher frequency of pedunculated (60.4%) and asymptomatic (84.7%) lesions. Also, the mean size of the lesions was 1.7 cm. Interestingly, we observed that, out of the 270 cases, 37 cases were larger than 2 cm. POFs that reach large proportions are referred to as huge/large, atypical, or gigantiform (23). It is believed that the increase of the diameter in these lesions occurs due to patients' fear of looking for dental care, as well as the lack of resistance to lesion proliferation in edentulous patients (25).

Due to its clinical similarity, POF performs differential diagnosis with other gingival nodular lesions, such as pyogenic granuloma and peripheral giant cell granuloma. Thus, requesting complementary exams is essential for POF's diagnosis (4). In this context, radiographic examinations may help in its diagnosis due to the possible presence of radiopaque foci in the soft tissue (13). In the present study, radiographic alterations were observed in 9.6% of the cases, in which focal or diffuse radiopacity was observed in 73.1%. Besides that, it is mandatory to send the specimen for microscopic analysis since pyogenic granuloma and peripheral giant cell granuloma exhibit different histopathological characteristics from those observed in POF. In the present study, it was observed that pyogenic granuloma was the most mentioned clinical hypothesis (45.6%). An interesting fact is that, although it has been suggested that POF may be a clinical progression of pyogenic granuloma maturation, no case showed a previous clinical history or microscopic features of pyogenic granuloma.

Regarding the histopathological features, the present study observed a higher frequency of lesions with hypercellularization of the mesenchymal component as well as the presence of inflammatory infiltrate, which was characterized as chronic and mild in most cases. Also, only 36.4% of cases show ulceration in the epithelium. In the analysis performed by Buchner and Hansen (11), mesenchymal hypercellularization was observed; besides, these authors reported that there may be variation in this cellularity, but that it generally is presented high. According to Shrestha *et al*. (6), it is believed that the mononuclear cells of the mesenchymal component originate from the periodontal ligament, and these cells may present osteoblastic, cementoblastic, or fibroblastic phenotype due to a metaplastic process caused by irritative factors such as dental calculus, orthodontic appliances, and irregular restorations. Besides that, the presence of irritative factors may justify our findings corresponding to inflammatory infiltrate and ulceration since, in reactional lesions, irritative factors are responsible for causing local, chronic, and low-intensity trauma, and consequently can lead to the presence of chronic inflammatory infiltrate and/or ulceration (26).

It is known that the main histopathological characteristic of POF is the presence of mineralized tissue within the connective tissue, which exhibits different patterns of deposition and variation in its quantity and distribution. Additionally, these deposition patterns are microscopically classified as mature or immature bone, cementum-like tissue, and dystrophic calcification (4,6,20). The present study showed a heterogeneous distribution and quantity of mineralized tissues in the analyzed cases, as well as that all lesions exhibited two or more types of these tissues. Similar to reported in the literature (4,6,20), we observed that mature and immature bone tissue were the most frequent in the lesions. When analyzing the types of mineralized tissues found in POF, Buchner and Hansen (11) observed that, in some cases, dystrophic calcifications acted as foci for the formation of osteoid tissue. It is important to highlight that these authors also reported that dystrophic calcifications and cementum-like tissue were frequently incorporated into the newly deposited bone tissue. In this context, they theorized this potential for the formation of mineralized tissues is inherent to periodontal tissue, and the presence of dystrophic calcifications may be an early morphological stage of POF maturation; thus, its presence promotes the formation of cementum-like tissue and immature and mature bone tissue.

Interestingly, we observed a significant association between high deposition of immature bone and size less than or equal to 1.7 cm, as well as a high deposition of cementum-like tissue and immature bone and evolution time equal to or less than 16 months. We think this result reinforces the theory of the spectrum of formation of mineralized tissues. The literature reports that the mean size and time of clinical evolution of POF is 2 cm and approximately 25 months, respectively (10,19,23,24). Thus, we believe POFs that exhibit size and evolution time lower than the reported average tend to exhibit mineralized tissues characterized as intermediate (cement-like tissue and immature bone) about the clinical maturation of the lesion.

Another significant finding of our study was the association between the hypercellularization of the mesenchymal component and the high deposition of immature bone. Additionally, likewise as observed by Buchner and Hansen (11), our study showed that ulcerated lesions significantly exhibited a higher quantity of dystrophic calcification. We believe these results are also related to the maturation stages of the lesion since Buchner and Hansen (11) reported that lesions in early clinical stages tend to exhibit ulceration and dystrophic calcifications, while lesions in advanced clinical stages exhibit more capacity for maturation of the mineralized tissues. Therefore, the presence of dystrophic calcification may indicate that early lesions are still unable to synthesize more complex mineralized tissues. In its turn, with the maturation of the lesion, there would be an increase in the quantity of metaplastic mononuclear mesenchymal cells and a higher deposition of cementum-like and immature and mature bone tissue around the dystrophic calcification. Another result that corroborates the histopathological maturation theory of the lesion was the negative correlation between cementum-like tissue and immature bone as well as the positive correlation between dystrophic calcification and cementum-like tissue; once that in early clinical stages, POF would exhibit a proportional deposition between dystrophic calcification and cementum-like tissue, while during the clinical course of the lesion, cementum-like tissue is being replaced by immature bone, which can be characterized as a complex mineralized tissue associated to an advanced stage of POF maturation.

The treatment of POFs that involve the anterior upper gingiva and reach great proportions is challenging. Its treatment can lead to aesthetic discomfort in the gingival area, tooth root exposure, post-surgical sensitivity, and interference in oral hygiene maintenance (7). The standard treatment for POF is conservative local resection. In this context, the excision of the lesion must go up to the periosteum; also, scaling and root planing must be performed to avoid recurrence, and irritative factors removal is essential. In addition, it is important to note that the excisional biopsy can result in mucogingival defects since it can involve all the adjacent keratinized tissue. Several surgical approaches such as subepithelial connective tissue graft, free gingival autograft, enamel matrix derivatives, guided tissue regeneration, and coronally advanced flaps can be used to try to augment the excised tissue (27). Other treatment modalities include the use of Nd:YAG laser or removal of the lesion with piezosurgery (27,28). The literature reports recurrence rates to range from 8% to 20% (4). In the present study, there was recurrence in 37 of the 270 cases analyzed. The high rates of recurrence are probably due to incomplete resections of the periosteum close to the pedicle of the lesion, as well as the non-removal of local irritative factors.

As the limitation of the present study, we can mention the absence of information regarding the identification and removal of possible irritative factors, as well as information about the treatment performed and its possible consequences, such as mucogingival defects. We can also mention the absence of information concerning the recurrence of the lesions, which were not present in most of the biopsy records used to collect the clinical information.

In summary, POF occurs more commonly in females, with a predilection for the anterior upper gingiva. It is associated mainly with incisors and exhibits a high frequency in the third and fourth decades of life. Due to its clinical similarity with other gingival reactive lesions such as pyogenic granuloma and peripheral giant cell granuloma, the biopsy is essential for the final diagnosis. The present study showed that there was a high frequency of hypercellularization of the mesenchymal component and a heterogeneous distribution and quantity of mineralized tissues in the analyzed cases. Besides that, our findings support the theory that the different mineralized tissues found microscopically in POF constitute a spectrum of maturation of this lesion.
